# Stress and Temperature Sensitivity of Photonic Crystals Resonant Cavity

**DOI:** 10.1155/2013/805470

**Published:** 2013-07-09

**Authors:** Yan Li, Xue Zhao, Xiao-li Li, Hai-wei Fu

**Affiliations:** School of Science, Xi'an Shiyou University, Xi'an, Shaanxi 710065, China

## Abstract

The temperature and stress characteristic of photonic band gap structure resonant cavities with square and graphite lattice have been studied by finite-difference time-domain method. The results show that the resonant cavities, both square and graphite lattice, have more and more resonant frequency with the cavity enlarging. And the curves between the resonant frequency and stress have better linearity. When the cavity enlarges enough, the curve between resonant frequency and temperature will become sectionalized line from nonlinear curve. Especially, the temperature sensitivity will be descending as the cavity is enlarging. Nevertheless, once some structures are put in the center of the cavity, the temperature sensitivity will be rising fast for this kind of cavity. Obviously, this character is convenient for us to achieve the specification measurement for temperature and stress.

## 1. Introduction

Since the concept, photonic crystals (PhCs), was put forward 20 years ago, it has obtained much attention. It was found that this kind of man-made material could confine and control electromagnetic wave on a scale comparable with the wavelength. At the same time, when we introduce a line or a point defect in PhCs, a mode of being suppressed in the lattice is localized. For this reason, the devices made by PhCs offer a wide range of applications such as antenna, filter, and wavelength division multiplex [1–3]. Recently, most of the energy was devoted in sensors made by PhCs due to their extreme miniaturization and integration. So far, many sensors based on PhCs technology have been proposed in the literature such as biomolecules detection sensors [4], quantum dots infrared sensors [5], and force sensors [6] and generated by employing nanofabrication. 

Temperature and stress sensor is a very important sensor because temperature and stress are the basic environment parameters in many fields such as bridge, high building, and dam, and in some areas such as chemical production and bioscience, temperature is the only parameter which must be monitored all the time. Nevertheless, with regard to this kind of sensor, it is a key technology for distinguishing and testing temperature and stress simultaneously, especially for the PhCs slab sensor, because temperature and stress often have effects, such as strain, elastooptical effect, thermo expansion effect, and thermooptic effect, on the sensor simultaneously. On the other hand, the resonant cavity is a key point in designing PhCs slab sensor, so it is very important for understanding the characteristic of the PhCs slab resonant cavity.

In this paper, we studied the photonic band-gap structure (PBGS) resonant cavity model in detail, which is made of GaAs pillars in air with square lattice and graphite lattice. As accounting for strain, elastooptical effect, thermoexpansion effect, and thermooptic effect, the variation of wavelength of the resonant cavity changing with force and temperature has been calculated by finite-difference time-domain (FDTD) method.

## 2. Theoretical Model

 After GaAs pillars grew from the substrate with square lattice or graphite lattice, we can cut a plane that is perpendicular to the GaAs pillars. Then a two-dimensional PBGS model made by GaAs pillars in air with square lattice or graphite lattice has been obtained. We use *ε*_*r*_ = 12.18 as the relative permittivity of GaAs [7].

We use the theory and parameters in the literature [8, 9] to calculate the resonant frequency of the PhC resonant cavity, which change with the temperature and stress. The main thought is as follows.

When electromagnetic wave spread in a nonmagnetic, nonconducting, linear and plane anisotropic medium, the components of electric and magnetic field satisfy the following Maxwell equations for TE mode

(1) $$\begin{array}{c} \frac{\partial E_{y}}{\partial z}=\mu_{0}\frac{\partial H_{x}}{\partial t},\;\;\frac{\partial E_{y}}{\partial x}=-\mu_{0}\frac{\partial H_{z}}{\partial t},\;\;\\ \frac{\partial H_{x}}{\partial z}-\frac{\partial H_{z}}{\partial x}=\frac{\partial D_{y}}{\partial t}. \end{array}$$

The constitutive equation for plane anisotropic medium is $\varepsilon_{0}\overset{⃑}{E}\;=\overset{⃡}{\beta }\;\;•\overset{⃑}{D}$, where $\overset{⃡}{\beta }$ is a dielectric impermeability tensor.

If strain was generated by thermal expansion due to variety of temperature, the strain and variety of temperature Δ*T* should satisfy the following equation:

(2) $$\begin{array}{c} S_{1}=S_{2}=S_{3}=\alpha \Delta T,\;\;S_{4}=S_{5}=S_{6}=0, \end{array}$$

where *S*_1_, *S*_2_, and *S*_3_ are the normal strain along *x*, *y*, and *z* direction, respectively, and *S*_4_, *S*_5_, and *S*_6_ are the shear strain. *α* is the thermal expansion coefficient of the material.

If strain was generated by stress, according to Hooke law, stress and strain should satisfy the following equation:

(3) $$\begin{array}{c} S_{M}=s_{MN}T_{N}\;\left(M,N=1,2,\ldots ,6\right), \end{array}$$

where *T*_*N*_ and *S*_*M*_ are 1 × 6 matrixes for stress and strain, respectively, *s*_*MN*_ is the elasticity obedience coefficient matrix.

There will be a photoelastic effect for the PhC as undergoing force application. The modification of dielectric impermeability tensor and stress meet with the following equation:

(4) $$\begin{array}{c} \Delta \beta_{M}=\underset{MN}{\prod }T_{N}\;\left(M,N=1,2,\ldots ,6\right), \end{array}$$

where ∏_*MN*_ is the piezooptical coefficient and Δ*β*_*M*_ is the modification of dielectric impermeability tensor.

And, there will be a photoelastic effect as undergoing variety of temperature application due to the thermal expansion. The modification of dielectric impermeability tensor Δ*β*_*M*_^(*T*)^ and strain satisfy the following equation:

(5) $$\begin{array}{c} \Delta \beta_{M}^{\left(T\right)}=P_{MN}S_{N}\;\left(M,N=1,2,\ldots ,6\right), \end{array}$$

where *P*_*MN*_ is the elastooptical coefficient.

The variety of temperature will also bring about thermooptic effect. If variety of temperature was Δ*T*, thermootic effect should satisfies the following equation:

(6) $$\begin{array}{c} \Delta {\overset{⃡}{\beta }}^{\left(O\right)}=\overset{⃡}{b}\Delta T, \end{array}$$

where $\overset{⃡}{b}$ is the thermooptic coefficient tensor, $\Delta {\overset{⃡}{\beta }}^{\left(O\right)}$ is the modification of dielectric impermeability tensor caused by thermooptic effect.

Based on the previous theory, we can investigate how stress and temperature influenced the resonant mode by FDTD method. The operation principle of the PBGS resonant cavity is based on the assumption as follows: (1) there is only normal stress acting on the cavity model along *x* direction, neglecting the shear stress action; (2) the main axis coordinate system of the material indicatrix of the resonant cavity is coincident with the coordinate in the following PBGS resonant cavity model; (3) the normal stress acting on the cavity model along *x* direction would change the site of the GaAs pillars, but the variety of the shape of the GaAs pillars and its elastooptical effect will be ignored, because the normal stress was thought acting on the substrate.

## 3. PBGS Resonant Cavity Formed with Square Lattice 

### 3.1. Cavity Design and Band Structure

**Figure 1:**
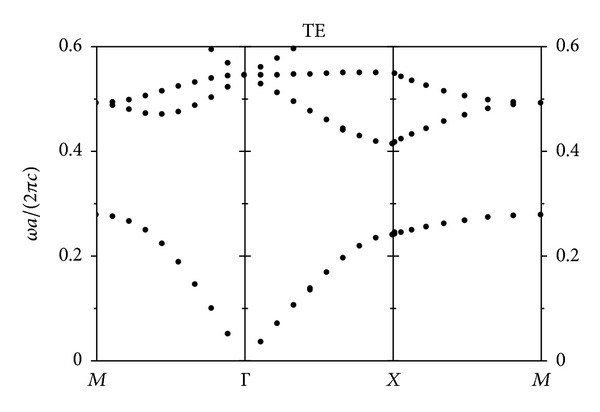
The TE mode photonic bands of two-dimensional photonic crystals made by GaAs pillars in air with square lattice.

Firstly, we have calculated the photonic bands of two-dimensional PhCs with square lattice for TE polarization, where the electric field component is parallel to GaAs pillars axis. The result is shown in Figure 1. 

The frequency unit in Figure 1 is *ωa*/(2*πc*), where a is the lattice constants of the PhCs with square lattice, *c* is the velocity of light in vacuum, and the radius of the GaAs pillars is *r* = 0.2*a*. It is found that there is a band gap between 0.2777 and 0.4135 in Figure 1 for TE mode.

**Figure 2:**
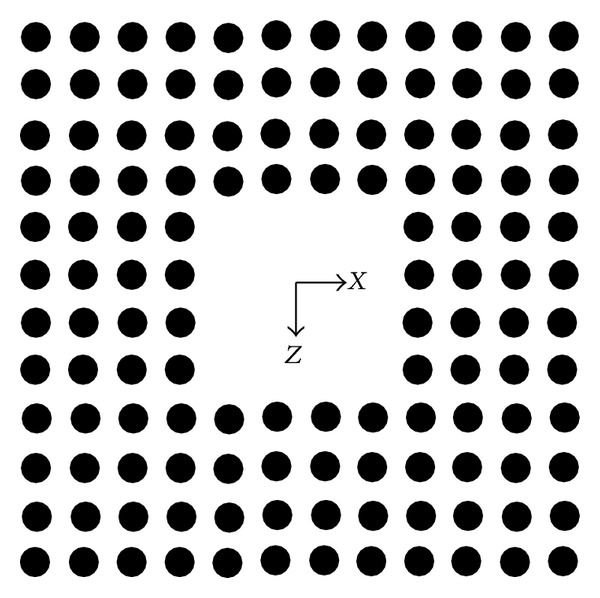
The 4 × 4 resonant cavity model of the two-dimensional PBGS made by GaAs pillars in air with square lattice.

 Based on the previous result, the two-dimensional PBGS made by GaAs pillars in air with square lattice, which has a band gap between 0.2777 and 0.4135, is put forward. After n rows and n columns of GaAs pillars in the center of the PBGS are removed, *n* × *n* PBGS resonant cavity model is given in Figure 2. 

When the resonant cavity has been excited by a modulated Gaussian pulse, where the driving source is put in the center of the cavity, the resonant frequency of the *n* × *n* cavity model is calculated by FDTD method in definite stress on *x* direction or temperature. 

### 3.2. Resonant Properties with Stress and Temperature

**Figure 3:**
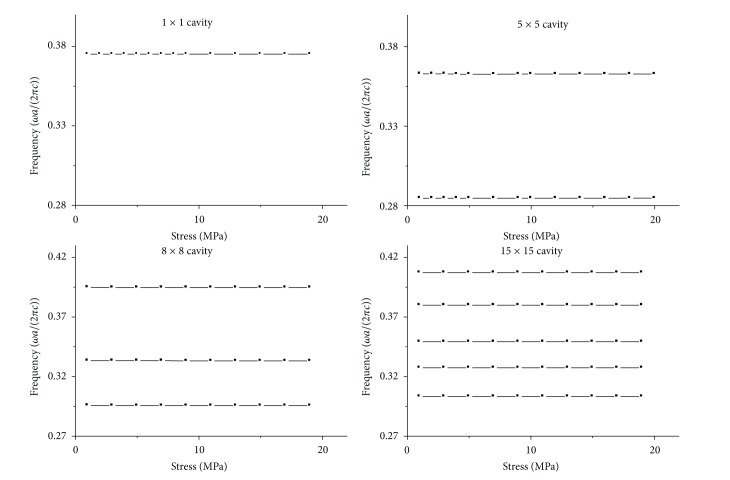
The resonant frequency for *n* × *n* cavity change with stress on *x* direction. 1 × 1, 5 × 5, 8 × 8, 15 × 15 represent cavities with *n* rows and *n* columns removed.

**Figure 4:**
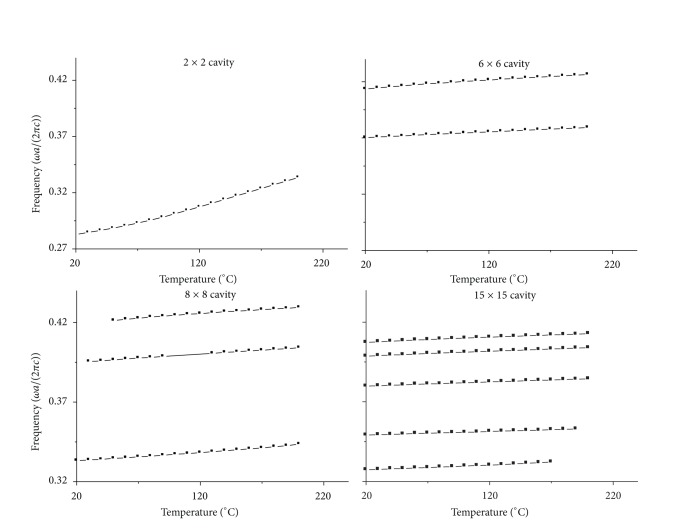
The resonant frequency for *n* × *n* cavity change with temperature. 2 × 2, 6 × 6, 8 × 8, 15 × 15 represent cavities with *n* rows and *n* columns removed.

From the simulation results, it is easy to find that the bigger the cavity is, the more resonant frequencies there are. This character is shown in Figures 3 and 4. 

Figures 3 and 4 show the results that the resonant frequency for *n* × *n* cavity changes with stress on *x* direction and temperature, respectively. 

The vertical axis in Figures 3 and 4 represents frequency, and its unit is *ωa*/(2*πc*). The horizontal axis in Figure 3 represents stress on *x* direction, where its unit is million pascal, and the horizontal axis in Figure 4 represents temperature, where its unit is degree centigrade. The principle for choosing the calculation result in Figures 3 and 4 is that the average normalized power spectrum is more than 20%.

From Figure 3 we can find that there are 1, 2, 3, 5 curves for 1 × 1, 5 × 5, 8 × 8, 15 × 15 cavity model, respectively, where the curves give the relation between resonant frequency and stress on *x* direction. And there are same curves for 2 × 2, 6 × 6, 8 × 8, 15 × 15 cavity model, respectively, in Figure 4, but the curves in Figure 4 give the relation between resonant frequency and temperature.

**Figure 5:**
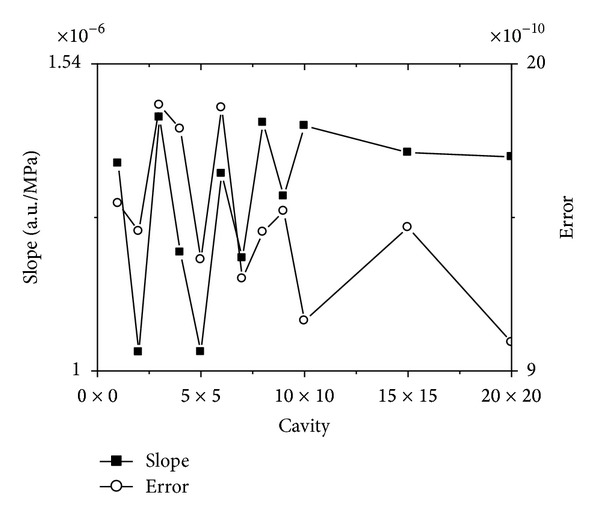
The slope and its error of the linear fitted curves for the resonant frequency changing with stress on *x* direction which has the biggest average normalized power spectrum.

Figure 3 also shows that it is linear relation between the resonant frequency and stress on *x* direction. To illustrate this case, we choose the curve with the biggest average normalized power spectrum from *n* × *n* cavity, and linear fit these curves. The slope and its error of the linear fitted lines are shown in Figure 5. 

The horizontal axis in Figure 5 represents *n* × *n* cavity. The left vertical axis in Figure 5 represents slope of the fitted line, and the right vertical axis in Figure 5 represents its error.

It can be found in Figure 5 that the error for every slope is very small, and the curve for error is gradually descending with the cavity enlarging.

**Figure 6:**
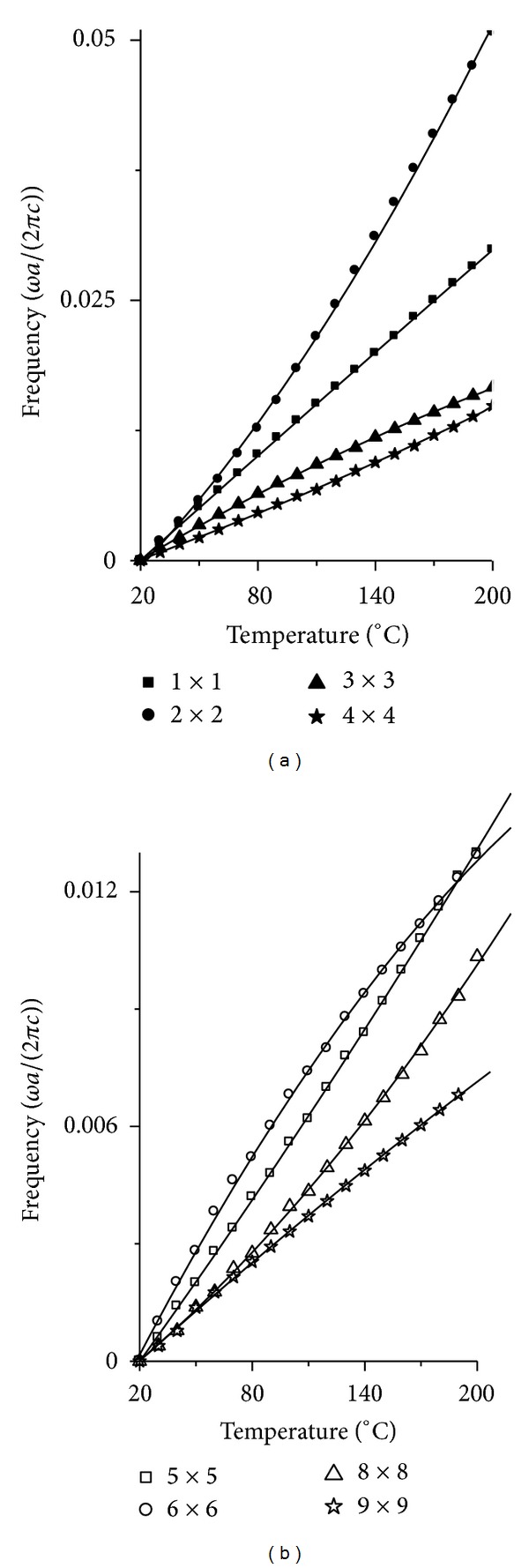
The shifted curves with the biggest average normalized power spectrum for *n* × *n* cavity, which give the relation between resonant frequency and temperature. The real lines in (a) and (b) represent the polynomial fit results. (a) is the result for 1 × 1 to 4 × 4 cavity. (b) is the result for 5 × 5, 6 × 6, 8 × 8, 9 × 9 cavity.

**Figure 7:**
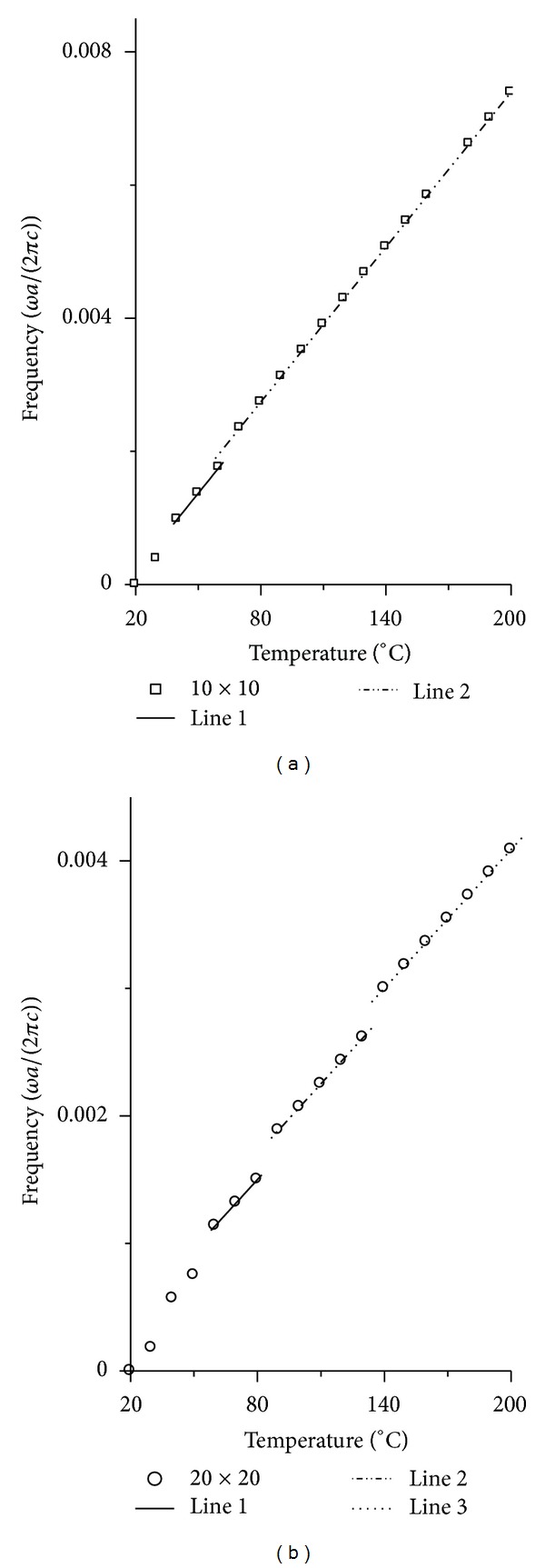
The shifted curves with the biggest average normalized power spectrum for *n* × *n* cavity, which give the relation between resonant frequency and temperature. The real lines, the dash dot line, and the dot line in (a) and (b) represent the line fit results. (a) is the result for 10 × 10 cavity. (b) is the result for 20 × 20 cavity.

However, when we contrast Figure 3 with Figure 4, we can find that the linearity is worse for the resonant frequency changing with temperature than that for changing with stress. The results in Figure 4 show that most of the curves are nonlinear, especially for 2 × 2 cavity in Figure 4. To reveal the nonlinear characteristic of the curves in Figure 4, we also choose the curves with the biggest average normalized power spectrum in Figure 4 and shifted them to 0 frequency at 20°C. At the same time, we fitted all the curves. The results are shown in Figures 6 and 7.

The vertical axes in Figures 6 and 7 represent frequency with unit *ωa*/(2*πc*), and the horizontal axis in Figures 6 and 7 represents temperature with unit degree centigrade. In Figure 6 the real lines represent the polynomial fit results, where the formula for fitting is *y* = *A* + *B*1 × *x* + *B*2 × *x* ^2^. But the real line, the dash dot line, and the dot line in Figure 7 represent the line fit results, where the fitted formula is *y* = *C* + *D* × *x*.

It is obvious for one to see the nonlinear character in Figure 6 for all the curves. But when we contrast Figures 6 and 7, we can find that the nonlinear character is weakening as the cavity is enlarging. When the cavity is large enough to 10 × 10, the curves become the sectionalized lines. 

**Figure 8:**
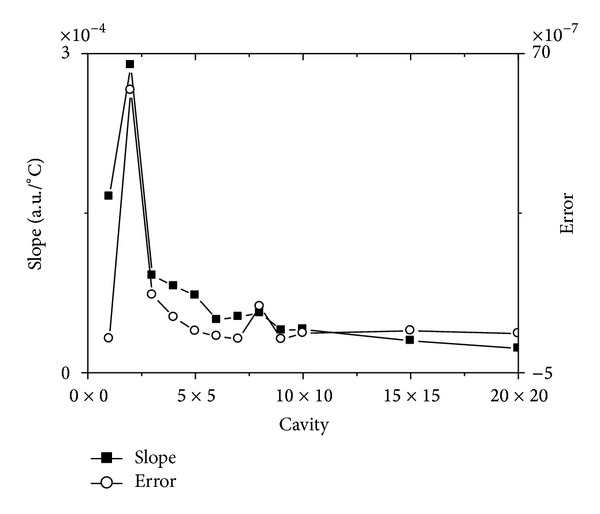
The slope and its error of the linear fitted curves for the resonant frequency changing with temperature which has the biggest average normalized power spectrum.

To further reveal the variety in Figures 6 and 7, we linear fitted all the curves in Figures 6 and 7 and gave their slope and error in Figure 8.

The horizontal axis in Figure 8 represents *n* × *n* cavity. The left vertical axis in Figure 8 represents slope of the fitted line, and the right vertical axis in Figure 8 represents its error.

 Contrasting Figure 5 with Figure 8, it is not difficult for one to see that the error in Figure 8 is bigger than that in Figure 5. But one can also find that there is a same tendency for Figure 8 with that for Figure 5. It means that not only the slope of the curves for Figure 8 is descending as the cavity enlarging, but also the error is reducing. Obviously, this case means that the bigger the resonant cavity is, the more linear the curves are. In another words, it means that the bigger the resonant cavity is, the more accurate for one to test the temperature.

On the other hand, one can find in Figures 6 and 7 that the bigger the cavity is, the smaller the slope of the curves is. This means that the sensitivity of the resonant cavity to temperature is descending when the square resonant cavity becomes more and more bigger.

### 3.3. Comparison of Resonant Properties between Stress and Temperature

**Figure 9:**
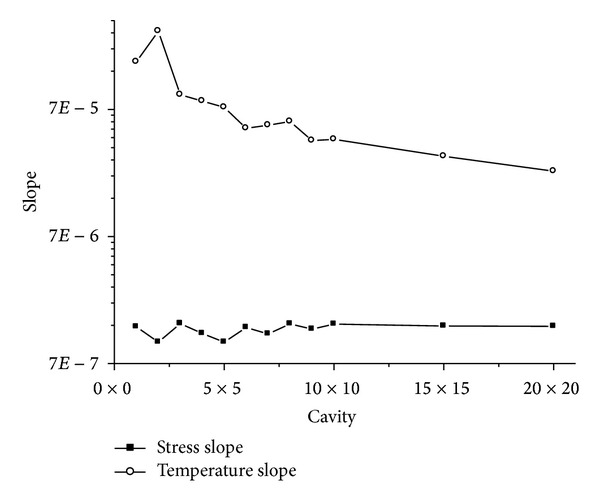
Slopes for the curve between resonant frequency and temperature and between resonant frequency and stress.

To illustrate the variation of the slope for the curves, we put the slope curve in Figures 5 and 8 together, and show them in Figure 9. The horizontal axis in Figure 9 represents *n* × *n* cavity. The vertical axis in Figure 9 represents slope of the linear fitted curves.

In Figure 9, one can find that the slope of the curves between resonant frequency and temperature is far bigger than that of the curves between resonant frequency and stress. This means that this kind of resonant cavity is more sensitive for temperature than that for stress. Obviously, it is a good sensor for testing temperature for this kind of PBGS slab cavity. And it can be used in testing the tiny variety of temperature due to its sensitivity. But in Figure 9, one can also find that the slope for temperature is descending when the resonant cavity enlarges. This means that we can reduce the sensitivity of the resonant cavity for temperature by enlarging the resonant cavity. 

**Figure 10:**
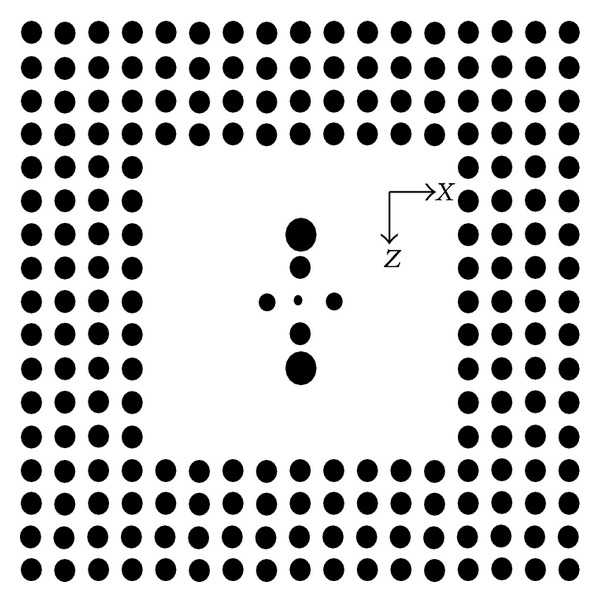
The two-dimensional PBGS 9 × 9 resonant cavity model with a cross structure in the center.

 However, when we add some structure in the center of the resonant cavity, such as the cross structure in Figure 10, the structure can adjust the temperature sensitivity of the resonant cavity effectively.

**Figure 11:**
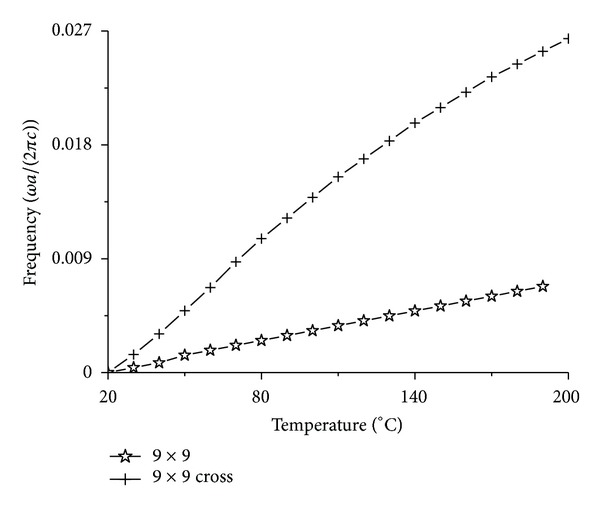
The parallel moved temperature curves with the biggest average normalized power spectrum for the 9 × 9 cavity and the 9 × 9 cavity with a cross structure in the center.

 The pillars in the center of the resonant cavity in Figure 10 are symmetrical distribution. The radius for the biggest pillar, the second biggest pillar, and the smallest pillar along *z* direction are *r*_2_ = 0.3*a*, *r*_1_ = 0.2*a*, and *r*_0_ = 0.1*a*. The radius of the biggest pillar along *x* direction is *r*_3_ = 0.15*a*. We have calculated the resonant frequency changing with temperature by the same method. The result is shown in Figure 11.

 The axes in Figure 11 are the same with that in Figure 6. The curve with legend 9 × 9 in Figure 11 is the same with that in Figure 6 completely. But the curve with legend 9 × 9 cross is one of the results for the resonant cavity in Figure 10, which has the biggest average normalized power spectrum. Contrasting the two curves, where two curves are all shifted to 0 frequency, one can find easily in Figure 11 that the temperature sensitivity for the curve with legend 9 × 9 cross is far bigger than that for the curve with legend 9 × 9.

 Based on the previous discussion, now we can give an explanation for the reason why the nonlinear character of the curves in Figure 6 is weakening with enlarging of the resonant cavity.

 On one hand, the parameter such as the thermooptic coefficient for GaAs is nonlinear. On the other hand, the relative variety of the shape of the resonant cavity, which is caused by temperature, will be reducing with enlarging of the resonant cavity, so the effect of the nonlinear character of the parameters on the curves will be weakening with enlarging of the resonant cavity. When the resonant cavity is big enough, the effect of the nonlinear character of the parameters on the curves will be ignored in some range. Then the nonlinear curves become sectionalized lines as in Figure 7.

## 4. PBGS Resonant Cavity Formed with Graphite Lattice

### 4.1. Cavity Design and Band Structure

Secondly, the temperature and stress characteristic of two-dimensional PBGS resonant cavity made by GaAs pillars in air with graphite lattice have also been studied with the same method.

**Figure 12:**
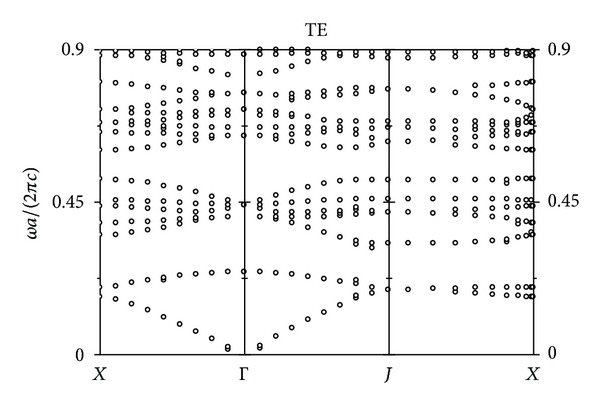
The TE mode photonic bands of two-dimensional photonic crystals made by GaAs pillars in air with graphite lattice.

Figure 12 shows the TE polarization photonic bands of two-dimensional PBGS made by GaAs pillars in air with graphite lattice. The frequency unit in Figure 12 is also *ωa*/(2*πc*), and the radius of the GaAs pillars is *r* = 0.25*a*. It can be found that there are three band gaps in Figure 12 for TE mode. The frequency intervals for these band gaps are [0.2431, 0.3277], [0.5180, 0.6026], and [0.8034, 0.8246], respectively.

**Figure 13:**
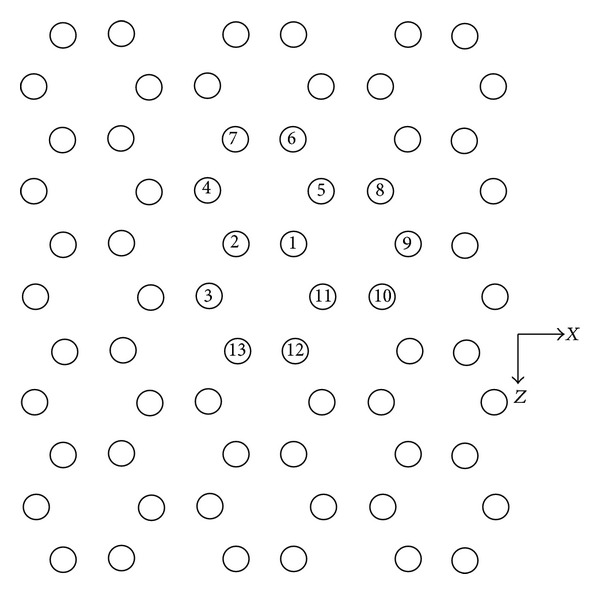
Resonant cavity model of the two-dimensional PBGS made by GaAs pillars in air with graphite lattice. The *n* cavity is put forward as No. 1 to No. *n* pillars removed.

The resonant cavity model of two-dimensional PBGS with graphite lattice is shown in Figure 13. When the GaAs pillars are removed from No. 1 to No. *n*, then a resonant cavity, which is called the *n* pillars cavity, is put forward. As the same above, when we put an impulse signal in the center of the cavity, the resonant frequency of the cavity can be calculated by FDTD method in definite stress on *x* direction or temperature.

### 4.2. Resonant Properties with Stress and Temperature

**Figure 14:**
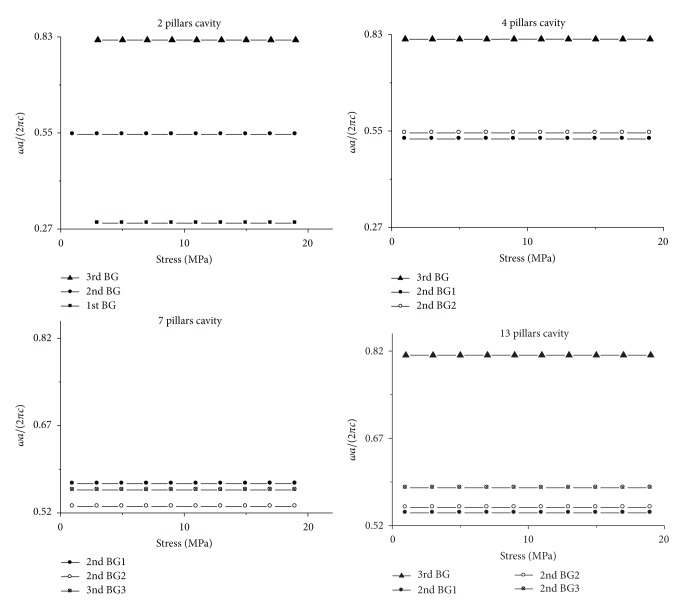
The resonant frequency change with stress on *x* direction for *n* pillars cavity. The four figures are the result for 2, 4, 7, 13 pillars cavity, respectively. The square, circle, and triangle legends represent the curves which are localized in the first, the second, and the third band gap, respectively.

**Figure 15:**
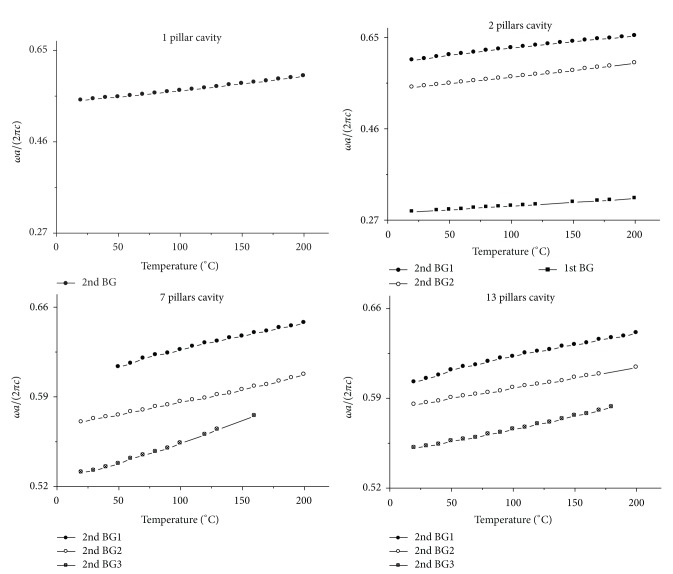
The resonant frequency change with temperature for *n* pillars cavity. The four figures are the result for 1, 2, 7, 13 pillars cavity, respectively. The square and circle legends represent the curves which are localized in the first and the second band gap, respectively.

The calculation results are shown in Figures 14 and 15. The vertical axes in Figures 14 and 15 represent frequency with unit *ωa*/(2*πc*). The horizontal axis in Figure 14 represents stress on *x* direction with unit million pascal, and the horizontal axis in Figure 15 represents temperature with unit degree centigrade. The principle for choosing the calculation result in Figures 14 and 15 is the same with that in Figures 3 and 4. In Figures 14 and 15, the square, circle, and triangle legends represent the curves that are localized in the first, the second, and the third band gap, respectively.

Figure 14 gives the relation between resonant frequency and stress on *x* direction. In Figure 14, one can find that there are three curves for 2 pillars cavity. Because these curves are localized in the first, the second, and the third band gap, respectively, so it can be called the single mode cavity. Likely, there are three curves for 4 and 7 pillars cavity, but they should be called two and three mode cavity, respectively, because two of the curves are localized in the second band gap, and the other is localized in the third band gap for 4 pillars cavity, as well as the three curves are all localized in the second band gap for 7 pillars cavity. Obviously, the 13 pillars cavity is a three-mode cavity, because three of the curves are localized in the second band gap and the other belongs to the third band gap.

Figure 15 gives the relation between resonant frequency and temperature. And the 1, 2, 7, and 13 pillars cavity in Figure 15 should be called single, double, and three-mode cavity due to the same previous reasons.

Contrasting Figures 14 and 15 with Figures 3 and 4, respectively, one can find that there are similar stress and temperature characters for the graphite resonant cavity with that for the square resonant cavity. On one hand, one can find in Figures 14 and 15 that the bigger the resonant cavity is, the more resonant frequency there is; on the other hand, it is linear relation between the resonant frequency and stress in Figure 14, and it is the nonlinear relation between resonant frequency and temperature in Figure 15.

**Figure 16:**
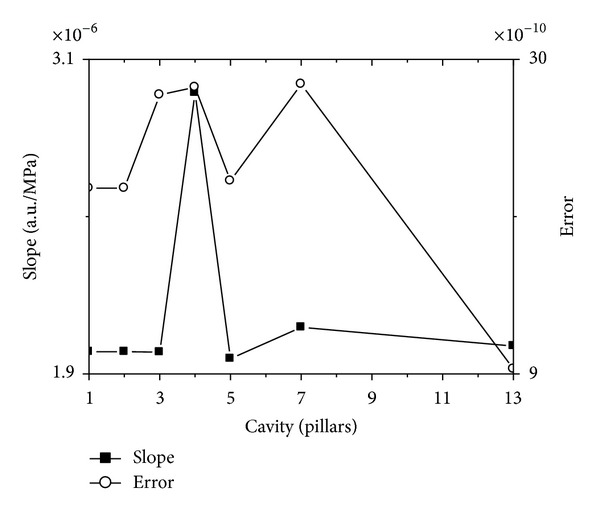
The slope and its error of the linear fitted curves for the resonant frequency, which has the biggest average normalized power spectrum, change with stress on *x* direction.

**Figure 17:**
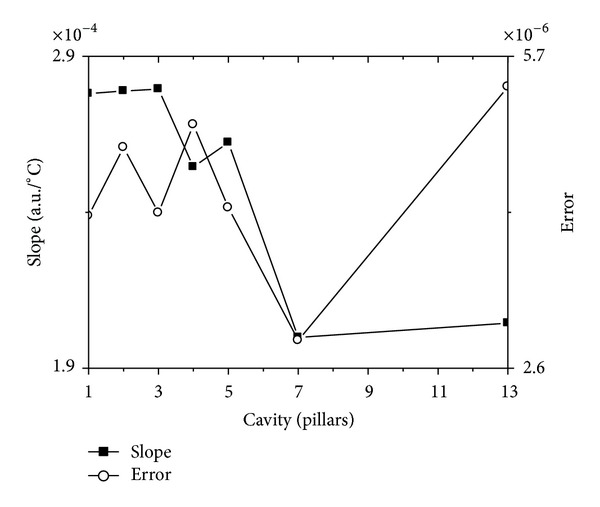
The slope and its error of the linear fitted curves for the resonant frequency, which has the biggest average normalized power spectrum, change with temperature.

To further illustrate the second character, we choose the same method like in illustrating the square lattice resonant cavity; it says that we choose the curve with the biggest average normalized power spectrum from *n* pillars cavity, and linear fit all the curves. The slope and its error of the linear fitted line for stress and temperature curves are shown in Figures 16 and 17, respectively.

The horizontal axes in Figures 16 and 17 represent cavity with unit pillars, and the left vertical axes in Figures 16 and 17 represent the slope of line fit result for stress and temperature curves, respectively, and the right vertical axes in Figures 16 and 17 represent their error.

Contrasting Figures 16 and 17, it is easy to find that the error of the slope in Figure 16 is far less than that in Figure 17. It says that the linearity for stress testing is better than that for temperature testing.

### 4.3. Comparison of Resonant Properties between Stress and Temperature

**Figure 18:**
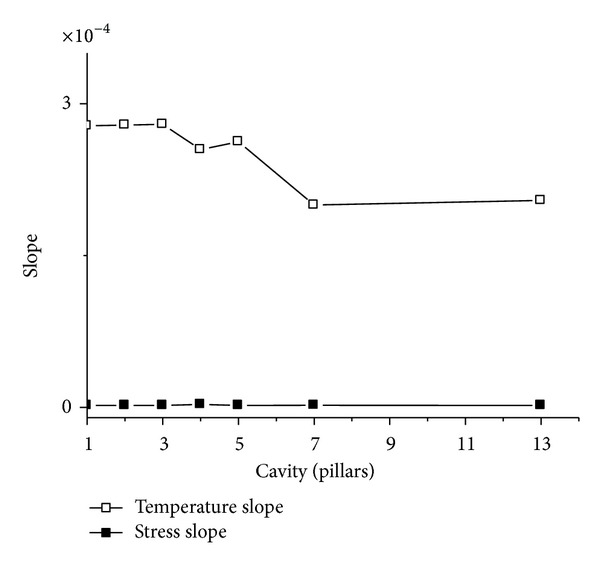
The slopes for the curves which are between resonant frequency and temperature and between resonant frequency and stress respectively.

When we put the slope curve in Figures 16 and 17 together, and show them in Figure 18, the similar character between graphite lattice resonant cavity and square lattice resonant cavity can be found. It says that the temperature slope is far bigger than the stress slope; it means that this kind of resonant cavity is more sensitive for temperature, and what is more, the slope for temperature is also descending when the resonant cavity enlarges. Of course, it means that we can reduce the sensitivity of the resonant cavity for temperature by enlarging the resonant cavity.

## 5. Conclusion

We have studied the temperature and stress characteristic of PBGS empty resonant cavities with square lattice and graphite lattice by FDTD method. The results show that the resonant cavities, both square and graphite lattice, have the similar character. Firstly, they have more and more resonant frequency with the cavity enlarging. Secondly, there is better linearity for the curves between the resonant frequency and stress. But when the cavity enlarges enough, the curve between resonant frequency and temperature will become sectionalized line from nonlinear curve. Obviously, this character is convenient for us to test temperature. At last, the most important character for the resonant cavities is that the slope of the curves between resonant frequency and temperature will be descending as the cavity is enlarging. It means that the temperature sensitivity will be descending as the cavity is enlarging. Nevertheless, once you put some structure in the center of the cavity, this kind of cavity will fast raise the temperature sensitivity. Obviously, this character is convenient for us to design the temperature and stress sensor.
